# Metabolic reprogramming and plasticity of cancer stem cells

**DOI:** 10.3389/fcell.2026.1807649

**Published:** 2026-05-29

**Authors:** Magdalena Kulus, Melania Marchel, Agnieszka Knopik-Skrocka, Krzysztof Data, Dominika Domagała, Weronika Bednarska, Paul Mozdziak, Bartosz Kempisty

**Affiliations:** 1 Department of Veterinary Surgery, Institute of Veterinary Medicine, Nicolaus Copernicus University in Torun, Torun, Poland; 2 Department of Cell Biology, Faculty of Biology, Adam Mickiewicz University of Poznan, Poznan, Poland; 3 Division of Anatomy, Department of Human Morphology and Embryology, Faculty of Medicine, Wroclaw Medical University, Wroclaw, Poland; 4 Prestage Department of Poultry Science, ⁠College of Agriculture and Life Sciences, North Carolina State University, Raleigh, NC, United States; 5 Physiology Graduate Faculty, North Carolina State University, Raleigh, NC, United States; 6 Department of Obstetrics and Gynecology, Center of Assisted Reproduction, University Hospital and Masaryk University, Brno, Czechia

**Keywords:** cancer stem cells’ plasticity, EMT, metabolic reprogramming, resistance to therapy, Tregs, tumor microenvironment

## Abstract

Numerous studies in cancer biology have provided evidence of the remarkable plasticity of cancer stem cells (CSCs). These cells play a key role in tumor initiation, metastasis, and resistance to treatment. One of the most critical features of CSCs is the epithelial–mesenchymal transition, which underlies their phenotypic plasticity. CSCs can modify their metabolic profile through interactions with cancer-associated fibroblasts, tumor-associated macrophages, and regulatory t-cells. CSCs creates a multifaceted cancerous environment that adapts to extensive changes in the secretome, variability of metabolic substrates and extracellular matrix composition. Intercellular communication is mediated though tumor-derived exosomes, carrying damage-associated molecular patterns. These metabolic shifts allow cancer cells to survive and function evading a hostile, immunosuppressive environment. It is important to summarize and integrate current knowledge on the links between cancer cell metabolism, CSCs plasticity, epithelial–mesenchymal transition, and immune regulation by regulatory T cells.

## 1 Introduction

The discovery of cancer stem cells (CSCs) marks a milestone in cancer research (Fanali et al., 2014; Knopik-Skrocka et al., 2024). Although the concepts and hypotheses surrounding the existence of these cells date back to the 20th century, when CSCs were first identified through research on acute myeloid leukemia (Bonnet and Dick, 1997; Lapidot et al., 1994). In subsequent years, CSCs were also identified in solid tumors, including breast cancer (Al-Hajj et al., 2003), glioma (Singh et al., 2004), colorectal cancer (Ricci-Vitiani et al., 2007), and they are known as a top of the hierarchy of cells composing tumor. According to the evolving framework of the “hallmarks of cancer,” malignant tumors exhibit numerous features essential for their growth and survival (Hanahan and Weinberg, 2000; Hanahan and Weinberg, 2011; Hanahan, 2022). Among these hallmarks, the extraordinary plasticity of CSCs encompass both phenotypic plasticity (linked to the epithelial–mesenchymal transition (EMT) and metabolic plasticity (Knopik-Skrocka et al., 2024). Recent studies have highlighted that CSCs plasticity results from both genetic and epigenetic changes, often mediated by non-coding RNAs (Bryl et al., 2022).

CSCs share several functional and metabolic features with normal tissue-resident stem cells, including self-renewal capacity, resistance to stress, and the ability to dynamically adapt their metabolism to environmental conditions (Psvv et al., 2026; Stouras et al., 2023). Normal stem cells, typically maintain metabolic flexibility to support quiescence, long-term maintenance, and rapid activation when required. For example, many adult stem cells rely on glycolysis and maintain relatively low mitochondrial activity, which helps limit reactive oxygen species (ROS) production and preserve genomic integrity (Khacho and Slack, 2018). However, upon activation or differentiation, these cells can shift toward increased mitochondrial oxidative phosphorylation (OXPHOS) and biosynthetic metabolism to meet energetic and anabolic demands (Shyh-Chang and Ng, 2017). CSCs appear to exploit similar metabolic programs but in a deregulated manner that supports tumor growth, survival under stress, and therapeutic resistance. Importantly, unlike normal stem cells that operate within tightly controlled physiological niches, CSCs often display enhanced metabolic plasticity, enabling them to dynamically switch between glycolysis, OXPHOS, and alternative metabolic pathways depending on environmental stimuli (Papadaki and Magklara, 2022). Thus, CSCs metabolism can be reprogrammed and exploited to support tumor initiation, progression, and resistance to therapy.

Due to their unique properties, CSCs may take a part not only for the initiation of carcinogenesis but also in subsequent development of the tumor, including metastasis, therapy resistance, and survival in hostile environments. Metabolic adaptation plays a crucial role in these processes, characterized by shifts in cellular metabolism between aerobic glycolysis (the Warburg effect) and OXPHOS, depending on the availability of oxygen, glucose, and metabolites such as lactate, specific amino acids, and fatty acids (FA) (Peiris-Pagès et al., 2016; Xie et al., 2022).

Heterogeneous population of CSCs is consisted of distinct functional states rather than representing a single, uniform cell type. Two classifications are frequently used to describe CSCs based upon the proliferative *versus* non-proliferative state or the epithelial-like *versus* mesenchymal-like phenotype. Importantly, these classifications are not mutually exclusive and likely represent interconnected dimensions of CSCs plasticity. Proliferating CSCs are typically characterized by active cell cycling and are thought to contribute primarily to tumor expansion, whereas non-proliferative, known as quiescent, CSCs display low proliferative activity, enhanced stress resistance, and are often associated with therapy resistance and long-term tumor persistence (Batlle and Clevers, 2017; Phi et al., 2018). EMT-linked division distinguishes epithelial-like CSCs, which are generally more proliferative and contribute predominantly to tumor growth, and mesenchymal-like CSCs, often more invasive, migratory, and associated with metastatic dissemination and therapy resistance (Celià-Terrassa and Jolly, 2020).

The force of CSCs plasticity is hypoxia and its regulator, HIF-1, which is responsible for metabolic reprogramming (Zhang et al., 2021). Years of research have established a strong relationship between CSCs metabolism and EMT (Zhang et al., 2021; Fedele et al., 2022). Proteins such as ZEB1, which plays a key role in EMT, can alter the expression of metabolic genes. The transition of CSCs from an epithelial-like to a mesenchymal-like phenotype is closely associated not only with changes in their metabolism but also, as a downstream effect, with the development of chemoresistance (Desbats et al., 2020; Lee et al., 2017). Furthermore, resistance to radiotherapy has also been documented as a consequence of metabolic modifications in cancer cells (Tang et al., 2018).

Several distinct metabolic patterns of cancer cells have been described to date (Naik and Decock, 2020; Peiris-Pagès et al., 2016). Among these pathways, non-stem cancer cells predominantly exhibit glycolytic activity, while CSCs favor either OXPHOS or a mixed glycolysis/OXPHOS pattern, depending on whether they are in a quiescent or proliferating state. A noticeable dominance of OXPHOS is typical in the quiescent state of CSCs, whereas a mixed metabolic pattern characterizes proliferating CSCs (Chae and Kim, 2018; Peiris-Pagès et al., 2016). Recently, research on the metabolism of cancer cells, including CSCs, has employed methods such as single-cell analysis using single-probe mass spectrometry (Sun and Yang, 2019). Studies on colorectal cancer cell lines (HCT-116) have shown significant differences in metabolism between CSCs and non-stem cancer cells. The levels of tricarboxylic acid (TCA) cycle metabolites, as well as unsaturated lipids and fatty acids, were significantly higher in CSCs compared to non-stem cancer cells. Furthermore, a positive correlation between pyruvate and fatty acid levels was demonstrated. Inhibition of aldehyde dehydrogenase (ALDH1A1) activity, a known marker of CSCs, leads to a decrease in unsaturated lipid levels and cancer stem cell properties. Research on the biology of CSCs is limited due to the insufficient number of these cells in experiments. The live single cell method therefore provides an opportunity to accurately understand the metabolome of CSCs.

The metabolic variability of CSCs is profoundly influenced by the tumor microenvironment (TME), with particular contributions from cancer-associated fibroblasts (CAFs) and immune cells (Zhu X. et al., 2020). CAFs differ from normal fibroblasts as they secrete growth factors, cytokines, extracellular matrix components, and metabolites that support tumor progression, especially CSCs metabolism (Lan et al., 2025). CAFs are directly involved in the so-called reverse Warburg effect (Zhu L. Y. et al., 2020), acting as an extracellular source of lactate for CSCs, which is utilized as a substrate in OXPHOS. Tumor-associated macrophages (TAMs) arise primarily from circulating monocytes or tissue-resident macrophages that are recruited and reprogrammed by tumor-derived signals. Once within the tumor, TAMs can influence multiple aspects of tumor metabolism, including immune regulation, angiogenesis, and metastasis (Zhu X. et al., 2020). Historically, macrophage activation has been described using the M1/M2 polarization paradigm, where classically activated M1 macrophages exhibit pro-inflammatory and anti-tumor properties. In contrast alternatively activated M2 macrophages are associated with tissue repair and pro-tumorigenic functions (Peng et al., 2025). However, this binary framework is now recognized as an oversimplification that does not adequately reflect the complex and dynamic phenotypic diversity of macrophages within tumors (Park et al., 2025). Increasing evidence from transcriptomic and single-cell analyses demonstrates that TAMs exist along a continuum of activation states shaped by local microenvironmental cues rather than discrete M1 or M2 categories. Classification approaches increasingly relying on combinations of surface markers, gene expression profiles, and functional characteristics (Strizova et al., 2023). TME cells appear to strongly interact with CSCs and can both promote tumor development and support the survival of cancer cells in an environment deficient in oxygen and nutrients (Naik and Decock, 2020; Pavlides et al., 2009). CSCs can also survive due to high lactate production and the metabolism of retinoic acid mediated by ALDH. Moreover, CSCs have also been shown to be activated by lactate (Gupta et al., 2022).

Among the immune cells infiltrating the TME and playing a significant role in cancer cell resistance, regulatory T cells (Tregs) stand out. These cells can adapt their metabolism to the prevailing environmental conditions (Kondo et al., 2024). Both Tregs and TAMs contribute to the induction of an immunosuppressive microenvironment (Mukherjee et al., 2023). The main threat posed by Tregs is their ability to inhibit the activity of cytotoxic T cells, for example, by disrupting the metabolism of these cells (Carmenate et al., 2018). Similar to CSCs, Tregs not only participate in tumor resistance to therapy but are also involved in the progression of cancer resistance to therapy (Dąbrowska et al., 2023; Kachikwu et al., 2011; Tang et al., 2018).

The objective of the manuscript is to consider the metabolic plasticity of CSCs and some of tumor microenvironment cells, such as s and CAFs, as key players in cancer metastasis and tumor resistance to therapy.

## 2 Metabolic reprogramming of CSCs

Dysregulation of cellular metabolism is one of the hallmark features of cancer cells (Hanahan and Weinberg, 2011). Metabolic reprogramming enables cells to produce energy in environments low in oxygen and nutrients, facilitating the survival and proliferation of cancer cells. The direction of these metabolic changes depends on the cancer cells phenotype and their state of activity (Luo and Wicha, 2015; Peiris-Pagès et al., 2016). Compared to non-stem cancer cells, CSCs tend to exhibit much higher plasticity. Non-stem cancer cells, which make up the bulk of the tumor, primarily rely on glycolysis, breaking down glucose into pyruvate followed by lactate. This results in significant acidification of the tumor microenvironment (Peiris-Pagès et al., 2016). This energy-acquiring mechanism is known as the Warburg effect, or aerobic glycolysis, because it mimics a process typically seen in anaerobic conditions, but occurs in cancer cells in the presence of oxygen (Zambrano et al., 2019).

### 2.1 Genes and epigenetic changes in metabolic reprogramming

**FIGURE 1:**
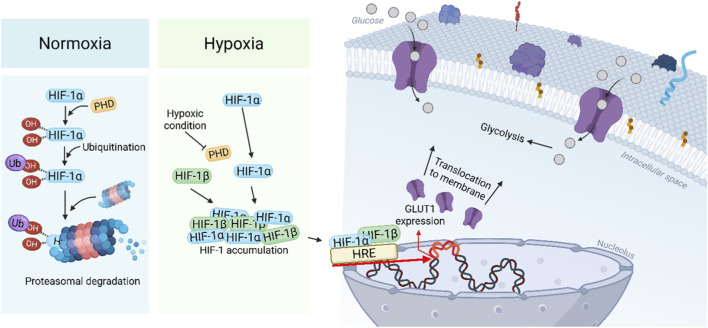
HIF-1 induced overexpression of GLUT1 related to hypoxic conditions (abb. HIF-1, Hypoxia-inducible factor-1; PHD, Prolyl Hydroxylase Domain; Ub, Ubiquitination; HRE, Hypoxia Responsive Element; GLUT1, Glucose Transporter Protein Type 1).

Cancer cells in hypoxia exhibit a high demand for glucose, which is associated with changes in the expression of the gene encoding the glucose transporter GLUT1 (Figure 1). GLUT1 overexpression serves as a compensatory mechanism for the lower efficiency of ATP synthesis compared to OXPHOS, supporting the Warburg effect. The increased expression of GLUT1, observed under hypoxic conditions, is driven by the activity of the HIF-1 complex, as a transcription factor (Zambrano et al., 2019; Zhang et al., 2022). GLUT1-driven metabolism additionally contributes to activation of stemness pathways, PI3K/AKT signaling pathway and mTOR signaling pathway (Zhou D. et al., 2020). GLUT1-mediated high glucose impact, specifically metabolic intermediates, are closely linked to epigenetic changes. Many of chromatin-modifying enzymes use metabolic intermediates as cofactors or substrates. When glucose uptake increases, the pentose phosphate pathway, and the TCA cycle generates metabolites that directly regulate epigenetic enzymes. Acetyl-CoA, one of the major metabolic intermediates, act as a substrate during histone acetylation, neutralizing lysine charge, chromatin loosening and increasing genes transcription (Chen et al., 2024; Hu et al., 2019). In CSCs, Acetyl-CoA may promote expression of stemness genes such as Oct4 and Nanog (Tsogtbaatar et al., 2020). Besides, other metabolic intermediates-linked epigenetic changes include influencing: S-adenosylmethionine on histone methylation, α-ketoglutarate on histone demethylation, NAD^+^ on histone deacetylation and lactate on histone lactylation (Wang and Han, 2022; Etchegaray and Mostoslavsky, 2016). Increased glycolysis, i.e., *via* GLUT1, and epigenetic changes, enhances therapy resistance (Niu et al., 2025).

The high glycolytic activity of cancer cells is associated also with increased Lactate dehydrogenase A (LDHA) expression (Wang et al., 2020), which has been observed in many types of cancer, including endometrial, pancreatic, and gastric cancers (Mishra and Banerjee, 2019). Increased glycolytic activity modulated by factors such as HIF-1, c-Myc, CREB, and FOXM1. LDHA can also be modulated by post-translational modifications, including phosphorylation and acetylation (Feng et al., 2018). Meta-analyses across various cancer types show that high LDHA expression is significantly correlated with the prognostic outcomes of cancer patients (Lv et al., 2019). Therefore, high LDHA levels are considered a poor prognostic factor. LDHA and aerobic glycolysis play critical roles in several hallmarks of cancer. In addition to promoting the proliferation and survival of cancer cells, this process can trigger angiogenesis *via* IL-8 upregulation. In human melanoma, LDHA expression is negatively correlated with T-cell anti-cancer activity, contributing to immunosuppression in the tumor microenvironment. (Feng et al., 2018).

**FIGURE 2:**
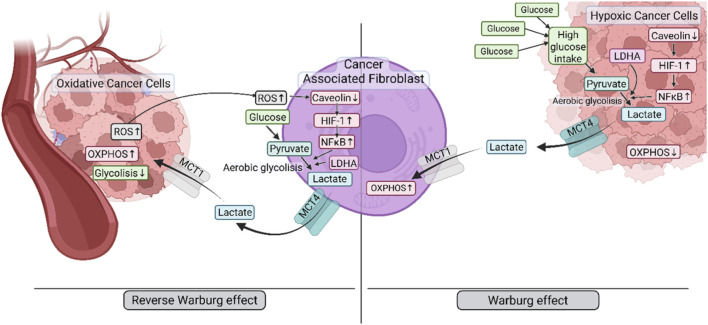
Reverse Warburg and Warburg effect in tumor microenvironment. (abb. ROS, Reactive Oxygen Species; OXPHOS, Oxidative Phosphorylation; HIF-1, Hypoxia-Induced factor 1; NFκB, nuclear factor kappa-light-chain-enhancer of activated B cells; LDHA, lactate dehydrogenase A; MCT1and4, Monocarboxylate transporter 1 and 4).

CSCs demonstrate the ability to increase OXPHOS efficiency by acquiring lactate from the TME, which is facilitated by significant cooperation with CAFs (Figure 2). These cells shift their metabolism to a glycolytic state, enabling lactate export into the microenvironment *via* the lactate monocarboxylate transporter (MCT). Subsequently, lactate is taken up by CSCs *via* MCT and converted to pyruvate (Naik and Decock, 2020). Pyruvate is then integrated into the Krebs cycle, which is referred to as the reverse Warburg effect (Pavlides et al., 2009). Under these conditions, LDHB expression is upregulated, allowing the conversion of lactate to pyruvate. Some malignant tumors exhibit high levels of LDHB (Mishra and Banerjee, 2019). For instance, KRAS-mutant lung cancers display elevated LDHB levels compared to KRAS wild-type cells. Similarly, LDHB is upregulated in triple-negative breast cancer (TNBC). In TNBC patients, MCT1 is co-expressed with LDHB, serving as a poor prognostic factor (McCleland et al., 2012).

### 2.2 Quiescent and proliferating CSCs subsets

Unlike non-stem cancer cells, CSCs can base their metabolism on aerobic glycolysis and OXPHOS, depending on the environmental conditions and activity of these cells (Peiris-Pagès et al., 2016; Xie et al., 2022). Regarding the differences in the metabolic phenotypes preferred by CSCs observed in different studies, it can be accepted that CSCs exist in multiple subsets, with the ability to choose/exhibit a particular metabolic phenotype (Thankamony et al., 2020). Proliferating CSCs and quiescent CSCs differ in metabolism, cell cycle activity, therapy response, and tumor-initiating capacity. Importantly, CSCs can transition between these states, which contributes to tumor heterogeneity and relapse.

Highly aggressive, proliferative CSCs are glycolysis-dominant, with high GLUT1-dependent glucose uptake. But also, are able to use, for example, FA (Fedele et al., 2022). Fatty acids oxidation (FAO) has been associated with progression and poor prognosis in patients with pancreatic cancer (Swierczynski et al., 2014). From the studies of Mascaraque et al. (2024), it can be assumed that FAO is critical for the tumorigenic potential and chemoresistance of pancreatic cancer stem cells. Interestingly, FA are not only synthesized inside the cell, but are also acquired by CSCs from cancer-associated adipocytes (CAAs). Fatty acid translocase (CD36) is involved in FA uptake and FAO in CSCs (Desbats et al., 2020; Nieman et al., 2011). The energy in quiescent CSCs is acquired often through OXPHOS. Proliferative CSCs can not only use glucose, but also, amino acids, fatty acids or ketone bodies (Xie et al., 2022).

According to data from De Francesco et al. (2018), the plastic transition from a quiescent to a proliferative state of CSCs relies on the metabolic shift from a glycolytic to an oxidative phenotype. Nevertheless, the ability to utilize both glycolysis and OXPHOS is also attributed to proliferating CSCs, which are defined as having a combined or mixed metabolic phenotype (Peiris-Pagès et al., 2016; Warrier et al., 2023). These cells exhibit high expression of both HIF-1 and AMPK (Table 1).

**TABLE 1:** Metabolic activity of cancer cells and its interplay with EMT.

Cancer cell activity	Metabolic activity	Glucose uptake	Functional and structural molecules biosynthesis	ROS level	Citations
CSCs quiescent	*OXPHOS* HIF-1^high^, AMPK^low^, MCT1^high^ (the reverse Warburg effect)	Lower glycolytic flux	Reduced biosynthesis	Very low	Peiris-Pagès et al. (2016)
CSCs proliferating	*Glycolytic* HIF-1^high^, AMPK^high^, MCT1^high^ (the reverse Warburg effect)	High; often GLUT1-dependent	High nucleotide, lipid and protein biosynthesis	Moderated	Jin et al. (2020)

Luo and Wicha, 2015, Knopik-Skrocka et al., 2024, Warrier et al., 2023.

CCs, non-stem cancer cells; CSCs, cancer stem cells; HIF-1, hypoxia inducible factor 1; AMPK, 5′AMP-activated protein kinase; MCT1 – monocarboxylate transporter 1; GLUT1 - Glucose Transporter 1.

AMPK acts as an energy and nutrient sensor, facilitating the restoration of ATP levels within cells. High AMPK activity in CSCs with a combined metabolic phenotype is associated with various metabolic processes, including the stimulation of glucose uptake and OXPHOS (Moldogazieva et al., 2020). There are interactions between HIF-1, glucose transporters, and AMPK with other regulatory proteins, including oncogenes such as c-Myc, p53, and KRAS protein, as well as growth factor-initiated pathways like protein kinase B (PKB/Akt), phosphatidylinositol-3-kinase (PI3K), and mTOR signaling. Additionally, high expression of MCT1 in proliferating CSCs, similar to quiescent cells, suggests the reverse Warburg effect on cellular metabolism.

Regarding lactate utilization by triple-negative breast cancer stem cells (TNBC) and their cooperation with the TME, four metabolic phenotypes have been proposed (Minami et al., 2021; Naik and Decock, 2020) (Table 2).

**TABLE 2:** Metabolic characters assigned to triple-negative breast CSCs.

Metabolic phenotype	Features	Citation
Warburg effect	Glycolytic TNBC cells with high level of the lactate transporter MCT4 export lactate, which is taken in by CAFs expressing MCT1 transporter to OXPHOS	Payen et al., 2020; Doyen et al., 2014
reverse Warburg effect	MCT4 expressing glycolytic CAFs export lactate to TNBC cells	Doyen et al., 2014; Choi et al., 2013
Mixed model	Glycolytic tumor cells and CAFs generate lactate to feed oxidative tumor cells	Naik and Decock (2020)
Hybrid model	TNBC cells switch between a glycolytic and oxidative phenotype	Jia et al. (2019)

Lactate is one of the metabolites that promotes cancer cell stemness (Table 3). A study on glioma demonstrated that lactate contributes to increased OXPHOS, aggressiveness, and stemness in a glucose-deprived microenvironment (Minami et al., 2021).

**TABLE 3:** The promotive effect of metabolites on stemness in different cancers.

Metabolite	Mechanism	Cancer	Citations
Glucose	OXPHOS → CSCs metabolic flexibility	Ovarian	Pastò et al. (2014)
Lactate	OXPHOS → aggressiveness and stemness	Glioma	Minami et al. (2021)
Glutamine	Redox homeostasis → ROS ↓ and Wnt/β-catenin↑ → stemness	NSCLC	Liao et al. (2017)
Glycine	Wnt-signaling → stemness	CRC	Terasaki et al. (2018)
Fatty acids	Fatty acid oxidation → CSCs survival and expansion	Ovarian	Tesfay et al. (2019)
Ketone body	Mitochondrial metabolism → CSCs stemness → tumor-initiating capacity	BC	Martinez-Outschoorn et al. (2012)

(Xie et al., 2022).

NSCLC, non-small cell lung cancer; CRC, colorectal cancer; BC, breast cancer.

### 2.3 Epithelial- and mesenchymal-like subtypes of CSCs and their metabolic pattern

**FIGURE 3:**
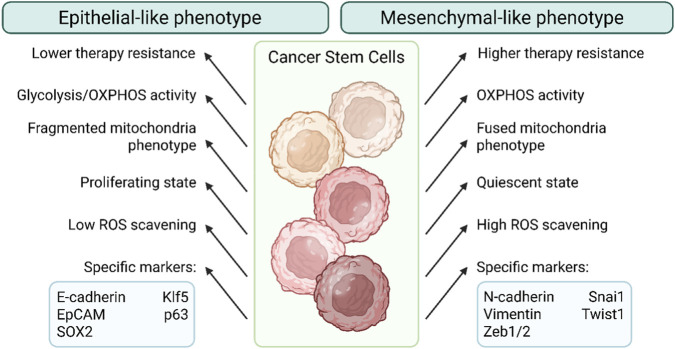
Link between CSCs metabolism and EMT. Changes in cell phenotype, metabolism, gene expression and transcription factors involved in EMT and therapy resistance (abb. OXPHOS, oxidative phosphorylation; EpCAM, Epithelial cell adhesion molecule; SOX2, SRY (sex determining region Y)-box 2; Klf5, Krueppel-like factor 5; p63, Tumor protein 63; Zeb1/2, Zinc-finger E-box-binding homeobox 1/2; Snai1, Snail Family Transcriptional Repressor 1; Twist1, Twist-related protein 1) (Gupta et al., 2019; Sun and Yang, 2020).

There is a strong association between the metabolic plasticity of CSCs and their ability to metastasize, which is conditioned by the EMT (Daniel et al., 2021). According to the metabostemness theory, metabolic reprogramming represents the initial step of EMT, making metabolic changes a significant driving force for CSCs. Regarding the origin theories of CSCs, non-CSCs can acquire stem-like features through metabolic alterations (De Francesco et al., 2018). Inhibition of OXPHOS has been shown to reduce sphere formation and tumorigenic potential, both hallmarks of the stem-like phenotype (Snyder et al., 2018). CSCs exhibit several key features in their epithelial-like and mesenchymal-like states (Sun and Yang, 2020; Gupta et al., 2019). Figure 3 highlights the most important of these characteristics.

The OXPHOS metabolic pattern is associated with quiescent CSCs, which exhibit a mesenchymal-like phenotype (Luo and Wicha, 2015). In contrast, epithelial-like CSCs are proliferative cells that significantly contribute to glycolysis. Enzymes involved in glycolysis, such as hexokinase 2 (HK2), phosphofructokinase (PFK), and pyruvate kinase, positively regulate glycolytic flux and induce EMT (Lai et al., 2020). In addition to the glycolytic switch, alterations in lipid metabolism also promote EMT and stemness (Daniel et al., 2021). Epithelial-like and mesenchymal-like CSCs possess unique lipidomic profiles (Giudetti et al., 2019). Studies on breast cancer cell lines have shown that the mesenchymal cell line MDA-231 has a higher percentage of saturated and polyunsaturated fatty acids compared to MCF-7 cells, which exhibit a higher percentage of monounsaturated FA. Furthermore, MDA-231 cells are characterized by a higher FAO than MCF-7 cells (Giudetti et al., 2019). Mitochondrial dysfunctions, including mutations in enzymes such as succinate dehydrogenase and the TCA cycle, also interact with EMT and stemness (Daniel et al., 2021). Metabolic plasticity may also arise from epigenetic changes in CSCs, such as histone methylation and acetylation (Perusina Lanfranca et al., 2019). This interplay between metabolism and epigenetics serves as an effective mechanism for CSCs to adapt to changes in nutrient and oxygen availability within the TME.

## 3 Contribution of TEXs on metabolic reprogramming in tumor microenvironment and therapy resistance

**FIGURE 4:**
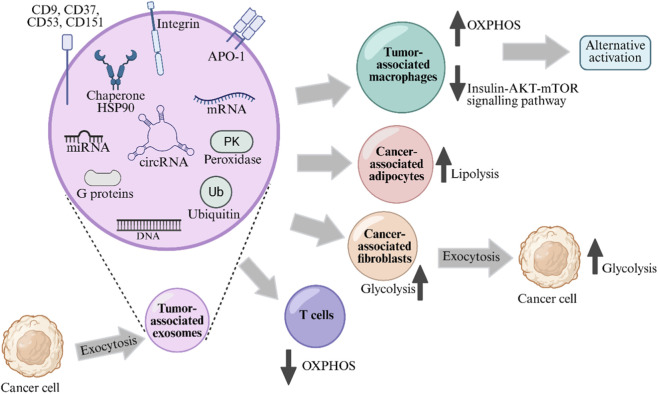
The impact of TEXs on tumor microenvironment (Fu et al., 2023; Hao et al., 2022, changed; abb. APO-1, Fas receptor; HSP90, Heat shock protein 90; OXPHOS, Oxidative phosphorylation; AKT, Protein kinase B; mTOR, mechanistic target of rapamycin).

CSCs secrete a specific type of extracellular vesicles, called TEXs (tumor-derived exosomes). They play a key role in creating an immunosuppressive TME, cancer resistance to therapy *via* metabolic reprogramming of cells, such as, e.g., CAFs, TAMs, MSCs and Tregs, as shown at Figure 4 (Fu et al., 2023, Morrissey et al., 2020). In CAFs and MSCs, there is a high level of glycolytic activity. miR-155 and miR-210 from melanoma TEXs are involved in decreased OXPHOS in fibroblasts (Shu et al., 2018). TEXs can reduce glycolysis in premetastatic niche, by reducing the expression of GLUT1 in non-tumor cells. As a result, a higher survival of metastatic tumor cells is possible. TEXs have the influence also on endothelial cells, increasing arginine metabolism and glycolysis, which promotes sprouting angiogenesis (Fridman et al., 2022). TEXs act on TAMs to promote OXPHOS and inhibit insulin-AKT-mTOR signal pathway, leading to cancerous plasticity. By acting on CAAs, TEXs promote lipolysis. TEXs impact on CAFs results in increased glycolysis but also leads to increased exocytosis. CAF-derived exosomes (CDEs) also promote glycolysis, through acting on cancer cell. However, exosomes produced by other cells of tumor microenvironment have influence on cancer cells. Under CAFs-derived exosomes, OXPHOS is decreased (Li Y. et al., 2020, Li C. et al., 2020). The inhibition of mitochondrial oxidative phosphorylation by CAFs-derived exosomes is associated with the increase of glycolysis (Figure 4).

Regarding tumor immunosuppression, one of their key mechanisms involves the regulation of immune checkpoint signaling, particularly the programmed cell death protein ligands 1 (PD-L1) pathway. TEXs are involved in the increase of immune PD-L1 checkpoints synthesis on macrophages or tumor cells. The interaction of PD-L1 with its receptors, expressed on T cytotoxic lymphocytes, triggers inhibitory signaling of T cells, leading to reduced decreased cytokines production, and impaired proliferation. Therefore, cytotoxic T cells lose their ability to effectively recognize and eliminate cancer cells. This immune checkpoint interaction therefore contributes to T-cell exhaustion and suppression of anti-tumor immune responses (Morrissey et al., 2020).

TEXs action results in metabolism deregulation and ATP production in T cells, such as T cytotoxic lymphocytes and T helper cells. The vesicles secreted by cancer cells can suppress the activity of T cytotoxic lymphocytes. These vesicles often express ectoenzymes such as CD39 and CD73, which convert extracellular ATP into adenosine in the tumor microenvironment. The accumulation of adenosine affects immune cells by binding to adenosine receptors on cytotoxic T lymphocytes. Activation of these receptors triggers intracellular signaling pathways that increase cyclic AMP levels in T cells, leading to inhibition of T-cell activation and effector functions. Therefore, the cytotoxic activity of T cells is significantly reduced (Sek et al., 2018). One of the consequences of this signaling is the decreased secretion of cytolytic molecules, including perforin, which is essential for killing tumor cells. Perforin normally forms pores in the membrane of target cells, allowing granzymes to enter and induce apoptosis. When adenosine signaling suppresses perforin release, anti-tumor immune response weakening (Tadokoro et al., 2020).

TEXs secreted by pancreatic cancer cells treated with gemcitabine promote chemoresistance *via* upregulation of the detoxifying enzymes of ROS and the decrease of gemcitabine conversion to its active metabolite (Patel et al., 2017). Via these mechanisms, enhanced antioxidant defenses and reduced drug activation, TEXs derived from gemcitabine-treated pancreatic cancer cells create a microenvironment that supports tumor cell survival and promotes chemoresistance.

Considering the important role of TEXs in the processes of CSCs cell function, they play a key role in cancer progression but also, may be a therapeutic target (Yang et al., 2020; Whiteside, 2016). There are several genetic and pharmacological strategies for TEXs inhibition. GW4869 is one of pharmacological inhibitor, used *in vitro* studies with MDA-MB-231 cells and T24 cells (Li et al., 2022). It seems important, in terms of overcoming resistance to cancer treatment, to link the typical signaling pathways of CSCs to their secretory activity.

## 4 CSCs resistance to therapy as a consequence of metabolic plasticity

Major fundaments of the cancer treatment are chemo- and radiotherapy, which are two strategies that aim to eliminate cancer cells using external stimuli, cytotoxic drug or high-energy radiation. The core differences is scope of treatment, targeted cellular state and main eliminating mechanism (Liu et al., 2021). Chemotherapy is a non-targeted, systemic therapy, often aimed at extensively proliferating cells. Cytotoxic drugs target rapidly dividing cells by interfering with DNA replication, mitosis, or metabolic pathways. Because CSCs may remain in a quiescent state, possess efficient drug efflux systems, and detoxifying enzymes, they are frequently less susceptible to chemotherapeutic agents. Radiotherapy is used locally, at the tumor site, damaging DNA of all the surrounding cells. To spare healthy tissues penetrated by radiation, such as skin, the radiation beams are directed at different angles to intersect with the tumor, thus providing a much higher absorbed dose at that site than in the surrounding healthy tissue. CSCs, however, tend to exhibit enhanced DNA damage repair capacity and stronger antioxidant defenses, which can reduce the effectiveness of radiation (Malik and Nie, 2012; Kim et al., 2023).

### 4.1 Chemoresistance and the role of CSCs’ quiescent state

Major component of drug resistance is metabolic plasticity, including changes in glucose metabolism (Wu et al., 2021). Alterations in glucose metabolism can facilitate the acquisition of drug resistance by enabling CSCs adaptation to metabolic stress and maintain survival under chemotherapeutic pressure. Identifying and understanding the intrinsic and extrinsic mechanisms of CSCs resistance are pivotal for effective anticancer therapy. A key feature of CSCs is metabolic plasticity, which enables them to dynamically adjust their energy metabolism in response to environmental, and treatment-induced stress. Switch between glycolysis and OXPHOS, depending on nutrient availability, oxygen levels, and metabolic demands, ensure an ATP and metabolic intermediates necessary for survival and proliferation (Kaur and Bhattacharyya, 2021).

Hypoxia-induced genes, such as ALDH, CD133, CD44, MDR1, and SIRT1, along with the activation of signaling pathways (e.g., Notch, PI3K/Akt, IL-6/STAT3, Wnt), are strongly involved in this process (Jiang et al., 2009; Knopik-Skrocka et al., 2024; Tripathi et al., 2023). Among CSCs markers, ALDH is widely recognized as a functional marker of stem-like cancer cells. Elevated ALDH activity is associated with the ability of cancer cells to form spheres, colonies, and initiate tumors, thus confirming the stemness phenotype of these cells (Clark and Palle, 2016). The enzyme activity promotes chemoresistance through enhanced DNA repair and reduced ROS levels (Clark and Palle, 2016) suggesting that ALDH plays a crucial role in cell detoxification and protection from oxidative stress. High resistance to cisplatin has been observed in lung cancer stem cells expressing high levels of ALDH (MacDonagh et al., 2017). ALDH is often found in combination with other CSCs markers, including CD133 and CD44. For example, tumorigenic potential is higher for CD133^+^ALDH^+^ cells compared to CD133^−^ALDH^−^ cells (Zhou et al., 2021).

One important mechanism contributing to therapy resistance is the dedifferentiation of cancer cells toward a CSCs or stem-like phenotype. Experimental dedifferentiation towards stemness can promote chemoresistance by increasing the IC_50_ (half maximal inhibitory concentration) dose of a chemotherapy drug by as much as 10-fold (Gupta et al., 2009; Gupta et al., 2019). This enhanced resistance reflects the ability of CSCs to activate survival pathways, increase metabolic adaptability, and efficiently repair cellular damage.

Finally, CSCs often exhibit increased activity of metabolic pathways involved in drug detoxification, including enzymes that metabolize xenobiotics and reactive metabolic by-products. These detoxification systems, such as glutathione S-transferases, cytochrome P450 enzymes, and ALDH isoforms, can inactivate chemotherapeutic agents or facilitate their elimination; thereby reducing intracellular drug toxicity and promoting chemoresistance (Mohan et al., 2021; Ramos et al., 2021). A positive correlation between the activity of the HIF-1α factor and tumor chemoresistance has also been described (Yong et al., 2022). HIF-1α activity contributes to the overexpression of efflux proteins, such as MRP1 or MDR1/P-glycoprotein. These proteins are responsible for pumping drugs out of the cell, as exemplified by the association between MDR1 overexpression and the resistance of breast cancer cells to taxanes and anthracyclines (Yong et al., 2022).

Quiescence is an important feature of CSCs-related resistance to therapy. Most cytotoxic chemotherapeutic agents are designed to target rapidly dividing cells, interfering with DNA replication, mitosis, or nucleotide synthesis (Lee et al., 2025a). CSCs that remain in a quiescent state avoid these processes because they are not actively cycling. Under these conditions, CSCs remain arrested in the G0/G1 phase of the cell cycle, and energy consumption is minimal (Liang and Kaufmann, 2023). EMT and metastasis processes favor CSCs with slow-cycling features (quiescence). CSCs in a quiescent state and exhibiting OXPHOS metabolism show much higher resistance to chemotherapy than proliferating CSCs (Lee et al., 2017; Chen et al., 2021). The quiescent state of CSCs is associated with lower levels of ROS. Many chemotherapeutic agents, such as 5-fluorouracil, cisplatin, and cytarabine, may induce quiescence in CSCs and promote chemoresistance (Chen et al., 2021). However, some chemotherapeutic agents can reactivate cancer cells and enhance their cytotoxic sensitivity to chemotherapy by promoting the transition of cancer cells from a quiescent state (Gao et al., 2017).

Mesenchymal-like CSCs, with quiescent feature, exhibit higher antioxidant capacity compared to the epithelial-like CSCs (Gammon et al., 2013). During the EMT process, cancer cells undergo changes in both phenotype and metabolism (Daniel et al., 2021). In the mesenchymal-like state, cancer cells acquire high resistance to therapy and enhanced invasion ability (Tripathi et al., 2023). CD133^+^ and CD44^+^ cancer cells exhibit high expression of ATP-binding cassette transporters, such as MDR1 (Li C. et al., 2020; Price et al., 2018). These markers are characteristic of stemness and are related to EMT capability (Gupta et al., 2009).

Therapeutic strategies that modulate CSCs metabolism and quiescence hold promise in the fight against CSCs. Numerous clinical trials are investigating new agents and metabolic modulators targeting, for example, mitochondrial complex I, MCT1, or IDH1 (Wu et al., 2021). Promising combination therapies involving chemotherapeutic agents and metabolic modulators are also being explored (Desbats et al., 2020).

### 4.2 Resistance to TKIs

Metabolic changes may contribute not only to chemotherapeutic resistance but also to resistance to targeted therapies using tyrosine kinase inhibitors (TKIs) (Wu et al., 2021; Zaal and Berkers, 2018). TKIs act by selectively blocking the kinase activity, thereby inhibiting phosphorylation-dependent signaling pathways. One of the most frequently described metabolic adaptations in TKIs resistance is the shift toward mitochondrial respiration. Resistant CSCs often develop enhanced mitochondrial OXPHOS, which supports survival during targeted therapy (Hirpara et al., 2019). Molecular alterations of EGFR, which is one of the TKIs target, are detected in 10%–35% of patients with non-small cell lung cancer (NSCLC). Consequently, several EGFR-targeting TKIs, such as gefitinib, erlotinib, afatinib, and osimertinib, have been approved for clinical use (Grodzka et al., 2023). Gefitinib-resistant cancer cells switch their metabolism from glycolysis to OXPHOS. Moreover, MCT-1 and LDHB are upregulated in these cells, indicating a reverse Warburg effect (Huang et al., 2020; Wu et al., 2021). This metabolic phenotype allows CSCs to maintain ATP production and sustain biosynthetic pathways even when oncogenic signaling pathways inhibited by TKIs are suppressed.

A novel mechanism of acquired resistance to EGFR TKIs, mediated by IGF2BP3-dependent cross-talk between epigenetic modifications and metabolic reprogramming was found in patients with NSCLC (Lin et al., 2023). Upregulated expression of the RNA binding protein IGF2BP3 in CSCs reduced sensitivity to TKI treatment *via* promoting OXPHOS.

Quiescence is an important feature of CSCs-related resistance to chemotherapy. Most cytotoxic chemotherapeutic agents are designed to target rapidly dividing cells, interfering with DNA replication, mitosis, or nucleotide synthesis (Lee et al., 2025a). CSCs that remain in a quiescent state avoid these processes because they are not actively cycling. Under these conditions, CSCs remain arrested in the G0/G1 phase of the cell cycle, and energy consumption is minimal (Liang and Kaufmann, 2023). EMT and metastasis processes favor CSCs with slow-cycling features (quiescence) (Mal, 2025). CSCs in a quiescent state and exhibiting OXPHOS metabolism show much higher resistance to chemotherapy than proliferating CSCs (Lee et al., 2017; Chen et al., 2021). The quiescent state of CSCs is associated with lower levels of ROS. Many chemotherapeutic agents, such as 5-fluorouracil, cisplatin, and cytarabine, may induce quiescence in CSCs and promote chemoresistance (Chen et al., 2021). However, some chemotherapeutic agents can reactivate cancer cells and enhance their cytotoxic sensitivity to chemotherapy by promoting the transition of cancer cells from a quiescent state (Gao et al., 2017). Mesenchymal-like CSCs (with quiescent feature) exhibit higher antioxidant capacity compared to the epithelial-like CSCs (Gammon et al., 2013). SIRT1 also promotes EMT and enhances MDR1 expression (Mvunta et al., 2017; Wang and Chen, 2013).

During the EMT process, cancer cells undergo changes in both phenotype and metabolism (Daniel et al., 2021). In the mesenchymal-like state, cancer cells acquire high resistance to therapy and enhanced invasion ability (Tripathi et al., 2023). CD133^+^ and CD44^+^ cancer cells exhibit high expression of ATP-binding cassette transporters, such as MDR1 (Li et al., 2017; Price et al., 2018). These markers are characteristic of stemness and are related to EMT capability (Gupta et al., 2009). Therapeutic strategies that modulate CSCs metabolism and quiescence hold promise in the fight against CSCs. Numerous clinical trials are investigating new agents and metabolic modulators targeting, for example, mitochondrial complex I, MCT1, or IDH1 (Wu et al., 2021). Promising combination therapies involving chemotherapeutic agents and metabolic modulators are also being explored (Desbats et al., 2020).

### 4.3 Radioresistance

**FIGURE 5:**
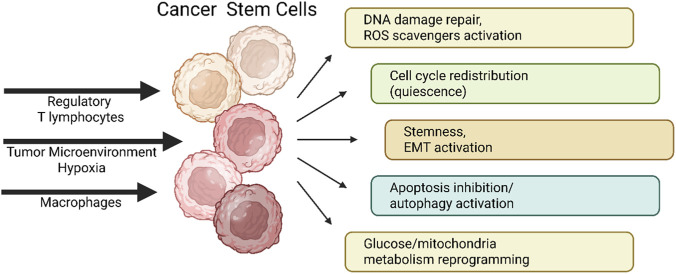
Different factors and mechanisms responsible for CSCs radioresistance (Tang et al., 2018, changed; abb. EMT, epithelial-mesenchymal transition; ROS, reactive oxygen species).

Radiotherapy directly causes DNA ionization and indirectly induces the production of ROS, which are normal byproducts of cellular processes. At low levels, ROS act as signaling molecules in cell communication. Moderate levels of ROS can enhance cancer cell metabolism and growth signaling (Nakamura and Takada, 2021). However, high levels of ROS can damage not only nucleic acids but also proteins, lipids, membranes, and even organelles. Radiotherapy is used in approximately 50% of cancer patients. Unfortunately, the effectiveness of this treatment is limited due to intrinsic or acquired radioresistance (Olivares-Urbano et al., 2020). Hypoxia strongly protects CSCs from ionizing radiation (IR) by inhibiting apoptosis and inducing autophagy. Figure 5 shows examples of factors responsible for CSCs radioresistance TME with different cells is engaged in this process.

As the chemo- and radiotherapy shares some of the ways of action, the resistance to therapy will exhibit some similarities. Both, chemo- and radiotherapy, will the mostly target the highly active cells, thus ability to switch into quiescent state, and the pathways that promote that switch, will be one of the universal factors of therapy resistance. Sun et al. (2015) demonstrated that under hypoxic conditions, HIF-1α induces autophagy, which decreases radiosensitivity in colon cancer cell lines. In hypoxia, irradiation promotes the CSCs quiescent phenotype and EMT (Lee et al., 2017; Zhou S. et al., 2020). While the majority of non-CSCs are eliminated, a small subset can acquire a stem-like phenotype (Li et al., 2016). The de-differentiation of non-stem cancer cells into CSCs supports the theoretical origin of CSCs from surrounding cells (Knopik-Skrocka et al., 2024). EMT-associated signaling pathways, EMT-inducing transcription factors, and EMT-related non-coding RNAs contribute to CSCs radioresistance. Activation of survival signaling pathways, such as Wnt-β-catenin, Notch, TGF-β, and PI3K/Akt/mTOR, has been observed in radioresistant CSCs (Olivares-Urbano et al., 2020; Schulz et al., 2019; Tanaka et al., 2019). Factors underlying CSCs resistance to IR include replication-associated DNA repair, enhanced activation of DNA damage checkpoints, and reduced cell proliferation, which drives CSCs into a quiescent state. Additionally, the high activity of detoxification systems in CSCs aids in the removal of free radicals. ROS scavengers play a critical role in this process (Rycaj and Tang, 2014).

Another universal, chemo- and radioresistant tool will be metabolic plasticity. The response of CSCs to radiotherapy involves changes in the expression of genes regulating mitochondrial function and metabolic pathways (Tang et al., 2018; Wu et al., 2023). Resistance to IR appears to be primarily dependent on glucose metabolism, though mitochondrial metabolism also plays a significant role in this process (Tang et al., 2018). Active glycolysis, HIF-1, elevated levels of GLUT1, and increased lactate production are hallmarks of radioresistant cancer cells (McCann et al., 2021; Quennet et al., 2006; Wu et al., 2023). Following IR, the abnormal expression of certain mitochondrial proteins and increased mitochondrial membrane potential contribute to DNA damage repair and inhibit cell apoptosis (Tang et al., 2018). Additionally, glutamine metabolism to glutamate may play a role in CSCs radioresistance, as inhibiting this process has been shown to sensitize these cells to radiotherapy (Li et al., 2015). Radioresistant cancer cells have also been observed to switch between glycolysis and oxidative phosphorylation, depending on cellular and microenvironmental conditions (McCann et al., 2021).

Mitochondria play a pivotal role in radioresistance, which is associated with changes not only in the mitochondrial energy metabolism profile but also in mitochondrial size and number. Consequently, certain drugs targeting mitochondria have the potential to act as radiosensitizers. The crosstalk between CSCs and the TME is a significant factor in the development of radioresistance. Recent studies have shown that factors secreted by CAFs, such as EGF, IGF2, and FGF4, contribute to the development of radioresistance (Chu et al., 2014; Suwa et al., 2021).

CXCL1 secreted by CAFs induces the accumulation of ROS in irradiated cancer cells by inhibiting the ROS-scavenging enzyme superoxide dismutase 1 (SOD1), thereby enhancing DNA damage (Zhang et al., 2017). However, in pancreatic cancer cells, increased activity of MnSOD has been associated with enhanced radioresistance (Tang et al., 2018). TAM also contribute to radioresistance and tumor regrowth after IR through the secretion of proangiogenic factors (Suwa et al., 2021). Lindström et al. (2017) demonstrated that hybrids of macrophages and MCF-7 breast cancer cells exhibit significantly higher radioresistance and enhanced DNA repair capacity compared to MCF-7 cells alone (Lindström et al., 2017). Among immune cells in the TME, Tregs also play a role in cancer radioresistance (Figure 5) (Ji et al., 2020). These cells, characterized by a CD4^+^, CD25^+^ (high), and FOXP3^+^ (high) phenotype, are attracted to the TME by chemokines such as CCL17 and CCL22, produced by tumor cells and other sources (Ketcham et al., 2018; Li C. et al., 2020). Irradiation increases the number of Tregs in the TME, and these cells exhibit higher resistance to IR compared to other immune cells (Kachikwu et al., 2011). The proliferation of Tregs following irradiation is facilitated by factors such as TGFβ or IL-33 (Jin et al., 2024).

## 5 Tregs metabolism and their contribution to tumor therapeutic resistance

Metabolites from immunosuppressive cells appear to support CSCs growth, survival, and therapeutic resistance through metabolic adaptations such as enhanced glycolysis and oxidative phosphorylation (Yu and Gong, 2025). Tregs actively participate in cancer metabolism by providing or promoting an environment rich in metabolites necessary for growth. These cells are strongly involved in immune suppressive microenvironment (Li Y. et al., 2020). Immune check points produced by Tregs, such as CTLA-4, are strongly associated with tumor immunosuppression (Li C. et al., 2020; Sobhani et al., 2021; Zhulai and Oleinik, 2022) and negatively impact the efficacy of immunotherapy (Bairi et al., 2021). Tregs recruitment and their immunosuppressive properties are influenced by hypoxia within the tumor microenvironment (Chen et al., 2023; Kondo et al., 2024).

Tregs can switch their metabolism (Table 4). Tregs that prefer lactate demonstrate stronger immunosuppressive and pro-neoplastic properties compared to those that prefer glucose (Watson et al., 2021). Unlike effector T cells, Tregs thrive in conditions with high lactic acid content. They transport lactic acid from the TME *via* MCT1, incorporate it into the TCA cycle, and subsequently upregulate PD-1 on their surface (Kondo et al., 2024; Liu et al., 2023).

**TABLE 4:** Metabolic plasticity of Tregs in TME.

Tregs activation	Metabolism	Citations
Proliferation	glycolysis^↑^, FAO^↑^, glutamine and arginine deficiency	Kishore et al., 2017; Gerriets et al., 2016; Klysz et al., 2015; Cobbold et al., 2009
Migration	glycolysis^↓^	Kishore et al. (2017)
Immunosuppression	glycolysis^↑^, OXPHOS^↑^, MCT1^↑^, FAO^↑^, FA^↑^, increased tryptophan metabolism	Miska et al., 2019; Yan et al., 2010; Haas et al., 2015

(Kempkes et al., 2019).

FAO, Fatty Acids Oxidation; FA, Fatty Acids; MCT1 - Monocarboxylate transporter 1.

Tregs have been shown to utilize different metabolic pathways depending on their phase of activity (Kempkes et al., 2019). Proliferating Tregs exhibit increased activity in both glycolysis and FAO. Although glycolysis is the predominant metabolic pathway for effector T cells, it plays a particularly crucial role in Tregs during proliferation and migration. Signaling pathways such as PI3K, MAPK, and mTOR are key drivers of glycolysis in Tregs, supported by GLUT1 expression (Kempkes et al., 2019). During proliferation, a deficiency in glutamine and tryptophan directs T cells to differentiate into Tregs.

Tregs’ ability to switch between glycolysis and FAO allows them to dynamically adapt to changing environmental conditions. Regulatory factors such as HIF-1α, mTOR, and AMPK play pivotal roles in controlling these metabolic processes. In their suppressive phase, Tregs exhibit reduced glycolysis alongside increased OXPHOS, FAO, fatty acid synthesis, and tryptophan metabolism. Shifting their metabolism toward OXPHOS enhances Tregs’ survival and immunosuppressive function (Kempkes et al., 2019). A key factor in the metabolic advantage of Tregs within the TME is the transcription factor FOXP3. FOXP3 inhibits the PI3K-Akt-mTOR pathway and suppresses Myc activity, thereby reducing glycolysis while promoting oxidative phosphorylation (Kondo et al., 2024).

In immunosuppressive state, Tregs have a direct effect on the activity and abundance of T effector cells in TME (Li Y. et al., 2020). This mechanism of action is based on the production and secretion of perforins and granzymes, which leads to the elimination of effector cells. The second mechanism involved in immunosuppression is the competition of Tregs with CD8^+^ for access to IL-2, which is essential for the proliferation of these cells (Carmenate et al., 2018). High expression of CD39 and CD73 on the surface of Tregs contributes to ATP hydrolysis, which generates free adenosine and ultimately inhibits effector cell proliferation *via* adenosine-adenosine receptor interactions A1, A2A, A2B, or A3 (Ohue and Nishikawa, 2019; Li C. et al., 2020). Another mechanism is based on indoleamine 2, 3-dioxygenase (IDO), the enzyme in the kynurenine pathway of tryptophan synthesis. This amino acid depletion in TME cause T effector cell dysfunction (Ohue and Nishikawa, 2019).

## 6 Therapeutic strategies and future perspectives

Anti-CSCs therapies poses a promising strategy in oncology due to their role in tumor initiation, therapy resistance, metastasis, and recurrence. Conventional treatments such as chemotherapy and radiotherapy primarily eliminate rapidly proliferating tumor cells but often fail to eliminate CSCs, especially the CSCs remaining in a quiescent state. As a result, surviving CSCs can repopulate the tumor after therapy (Stouras et al., 2023).

Actually, there is no therapeutic tool that can find, identify and eliminate specifically CSCs, thus anti-CSCs strategies may target the stemness-, EMT- and metabolic plasticity-related signaling pathways (Milella et al., 2025). However, the features that characterize CSCs are also those that enable them to efficiently avoid current therapeutic tools. Heterogeneity and plasticity causes that the CSCs population is not homogeneous; different CSCs at the same tumor exhibit various gene expression profiles, metabolic interactions, and responses to therapy, making up overall tumor heterogeneity and therapy resistance. At the same tumor site, CSCs can synthetize various drug efflux transporters, DNA repair enzymes, and anti-apoptotic proteins, allowing them to survive anticancer therapy (Heppner et al., 1978). Another major challenge in anti-CSCs therapy is the metabolic of CSCs. Their metabolic plasticity allows them to survive under hostile conditions, evade metabolic inhibition and therapy-induced cell death. A key component of this flexibility is the capacity of CSCs to reprogram ATP production in response to metabolic targeting. To enable CSCs-targeted therapy, specific biomarkers are necessary, what remains a significant challenge for both CSCs research and therapeutic targeting. Several markers, including CD44, CD133, ALDH1 and EpCAM, have been widely used to detect and isolate CSCs populations across different cancer types. However, these markers are not universally expressed and are frequently shared with non-CSCs, raising concerns about their specificity and potential off-target effects (Lee et al., 2025a). The crucial therapeutic target is to target the process of CSCs phenotypic change.

### 6.1 Stem cells signaling pathways inhibitors

One of the most frequently studied strategies is the inhibition of key signaling pathways responsible for maintaining CSCs stemness, especially embryonic stem cells markers, such as OCT-4, Nanog, Notch, and Wnt/β-catenin. These pathways regulate CSCs self-renewal, differentiation, and survival. These strategies demonstrate high *in vitro* efficacy, but have no clinical translation (Lee et al., 2025a). During research of stem-like properties in lung cancer cells, Chen et al. (2008) revealed that knock-down of OCT-4 expression can significantly inhibit tumor metastatic and colony formation. Another study employing prostatic cancer cell lines, and primary tumors suggested that Nanog inhibitors suppress the formation of spheroids from primary prostate cancer cells, clonal growth, and tumorigenesis (Jeter et al., 2009). Furthermore, Acikgoz et al. (2025), suppressed the Notch signaling pathway to eliminate CSCs population from tumor cells. Also on hepatocellular carcinoma, targeting Nanog, OCT4 and Wnt/β-catening pathway suppress stemness of the tumor cells (Jiang et al., 2025). However, these successful results have been demonstrated under controlled *in vitro* culture conditions.

### 6.2 Immunotherapy

CSCs often express specific surface antigens (e.g., CD44, CD133, EpCAM) that can be used in immunotherapy. Modified immune cells, like chimeric antigen receptor cells (CAR), especially CAR T-cell (CAR-T), CAR natural killer cells (CAR-NK), and CAR-macrophages (CAR-M), may offer another therapeutic strategy. CAR constructs typically comprise an extracellular antigen-binding domain, a transmembrane domain, and an intracellular signaling domain. CAR-T cells require specific surface markers, such as CD133 (clinical trial NCT02541370; Feng et al., 2018), to identify target cells. It appears that CAR-T targeting CD133 against cholangiocarcinoma may be feasible (Feng et al., 2018). Furthermore, CD-133 has been used with the CAR-NK platform to efficiently treat ovarian (Klapdor et al., 2017). CAR-NK aimed against EpCAM also efficiently suppress CSCs proliferation in colorectal tumors (Beard et al., 2014). Another population, CAR-M, targeted on CSCs-specific markers, exhibit increase phagocytic activity, pro-inflammatory cytokines release and finally elimination of CSCs in various tumors (Morva, et al., 2025; Hadiloo et al., 2023). A major advantage of this solution may be immunological memory, which can constitute an effective systemic anti-cancer barrier. However, the TEM, as well as the CSCs, may suppresses immune activity. Another challenge is that CSCs may exhibit variability in surface biomarkers indicating that targeting specific markers, although effective in research (testing for a specific type of marker), may leave individual subpopulations of CSCs in the TME.

### 6.3 Metabolic-related therapeutic strategies

Targeting metabolic pathways during cancer therapy is a promising strategy for eliminating CSCs, whose unique feature is their exceptional metabolic plasticity. Their ability to dynamically switch between various metabolic pathways, such as glycolysis, OXPHOS, glutamine metabolism, and FAO, makes them untargetable in conventional metabolic therapies. The metabolic adaptability of CSCs allows them to survive in the unfavorable conditions of the TME and in the presence of cytotoxic drugs, increasing their resistance to conventional therapies.

One of the most studied drugs targeting CSCs metabolism is metformin, which is commonly used to treat type 2 diabetes (Lv et al., 2025). Metformin affects mitochondrial metabolism by inhibiting complex I of the respiratory chain, leading to reduced OXPHOS and ATP production. Numerous preclinical studies have demonstrated that metformin can selectively reduce CSCs populations, including in colorectal and pancreatic cancer (Deng et al., 2020; Teper et al., 2023). In colorectal cancer, metformin not only reduces OXPHOS, but also suppress capacities to form spheres, colonies, proliferation and stemness- and differentiation-related genes, as CD133, c-MYC, MUC2, FABP2 and Wnt pathway (Yan et al., 2023). Besides, metformin increases chemosensitivity *via* downregulation DNA replication machinery (Kim et al., 2017). Ning et al. (2016) confirmed direct metformin effect on CSCs. By culturing pancreatic cancer spheroids, demonstrated that with the higher expression of CSCs surface markers in cell culture, the chemoresistance grows and the sensitivity to metformin increase.

Another important strategy is the inhibition of glutamine metabolism, which plays a key role in maintaining redox homeostasis and supplying metabolites to the TCA. CSCs often have an increased demand for glutamine, which is a reason that glutaminase inhibitors, such as CB-839 (telaglenastat) are being intensively investigated as potential therapeutic agents (Poonaki et al., 2022; Kahya et al., 2025). Inhibiting glutaminolysis leads to redox imbalance, reduced nucleotide biosynthesis, and decreased energy production, which can significantly limit the survival and self-renewal capacity of CSCs. Prostate CSCs can become radiosensitive in effect of drug-derived glutamine deprivation. Therefore, the combination of targeted glutamine metabolism leads to support radiosensitivity of prostate cancer (Mukha et al., 2021). In addition to cell death, a reduction in stemness capacity has also been demonstrated, primarily due to Wnt/β-catenin blockade (Li et al., 2019).

Another important therapeutic target eliminate CSCs is lipid metabolism, particularly FAO. In many types of cancer, CSCs have been shown to utilize FAO as an alternative energy source, particularly under conditions of nutrient deprivation or metabolic stress (Lee et al., 2025a). Therefore, FAO appears particularly relevant to the quiescent population of CSCs that are so elusive to other types of classical therapy. Chimge et al. (2024) used an FAO inhibitor to efficiently eliminate the quiescent state, treatment-resistant CSCs to sensitize them to imatinib, cytostatic chemotherapeutics. FAO inhibitors may therefore limit the adaptive capacity of CSCs, leading to decreased ATP production and increased susceptibility to oxidative stress (Mascaraque et al., 2024). Blocking this metabolic pathway has shown promising results in preclinical models, especially when combined with other metabolic therapies.

In CSCs metabolic plasticity studies, CRISPR/Cas9 method can be very helpful. CRISPR/Cas9-based synthetic lethality screens have been used to identify CSCs-specific metabolic vulnerabilities, leading to the development of novel combination therapies targeting both CSCs survival pathways and metabolic dependencies. By integrating genomics, transcriptomics, proteomics, metabolomics, and lipidomics data, machine learning algorithms can identify CSCs-specific vulnerabilities and optimize personalized therapeutic strategies (Lee et al., 2025b). As the Kanaer et al. provided, alpha-fetoprotein (AFP) CRISPR/Cas9-mediated gene knockout efficiently reduces CSCs stemness, downregulating pathways related to EpCAM, CD44 and PI3K/Akt signaling (Kahaer et al., 2026). The combination of CRISPR functional genomics with single-cell multiomics is expected to further refine our understanding of the biology and therapeutic vulnerabilities of CSCs. Single-cell transcriptome analysis may uncover intratumoral heterogeneity, as proven on hepatobiliary tumor, indicating drug resistance-related genes in a complex gene expression network, as catenin beta-1, NEAT1 (nuclear paraspeckle assembly transcript 1), NDRG1 (N-Myc downstream regulated 1), ALDOA and CA9 (Zhao et al., 2021). Single-cell analysis consist also a tool for evaluating CSCs and CAFs response to different treatments, ensuring proper and precisely selected therapy parameters (Lee et al., 2021).

Although many metabolic strategies demonstrate high efficacy in experimental models, their systemic clinical effectiveness and may be limited by the ability of CSCs to switch between different energy sources. Therefore, increasing attention is being paid to combination therapies that simultaneously inhibit multiple metabolic pathways or combine metabolic inhibitors with chemotherapy or immunotherapy, which may increase the effectiveness of CSCs elimination.

## 7 Conclusion

Intensive research into the metabolic plasticity of CSCs and their microenvironment has revealed the extraordinary complexity of this phenomenon, which underlies tumor resistance to therapy. CSCs exhibit an adaptive capacity in terms of ATP production and the choice of metabolic substrates, a process known as metabolic reprogramming. There is a close relationship between metabolic plasticity, EMT, and the proliferation status of CSCs. Interactions between CSCs and selected components of the tumor microenvironment, such as CAFs, TAMs, and T cells, play a crucial role in CSCs survival. One of the key mechanisms of this communication involves TEXs and other extracellular vesicles released into the TME.

Tregs, recruited by cancer cells, play a particularly important role in establishing an immunosuppressive microenvironment and, consequently, in therapy resistance. Their cellular metabolism governs their generation, proliferation, and suppressive function. Depending on conditions, Tregs can alter their metabolism and influence the activity of other cells. Inhibiting Tregs metabolic plasticity, including tryptophan metabolism, may reduce their immunosuppressive activity.

Both genomic and metabolomic profiling of tumors can aid in selecting effective therapeutic strategies. By identifying key metabolic pathways and their regulators in CSCs and associated cells, it will be possible to disrupt these processes. Combining therapies, especially metabolic, that target the metabolic plasticity of CSCs, Tregs, and other cells with immunotherapy and radiotherapy and cytotoxics, may enhance overall treatment efficacy. However, future research should focus on the selective delivery, targeted action of new therapeutic agents minimizing the side effects of treatment.
